# Inter-Relationships Between the Deep Learning-Based Pachychoroid Index and Clinical Features Associated with Neovascular Age-Related Macular Degeneration

**DOI:** 10.3390/jcm14093245

**Published:** 2025-05-07

**Authors:** Michiyuki Saito, Mizuho Mitamura, Yuki Ito, Hiroaki Endo, Satoshi Katsuta, Susumu Ishida

**Affiliations:** 1 Department of Ophthalmology, Faculty of Medicine and Graduate School of Medicine, Hokkaido University, N-15, W-7, Kita-ku, Sapporo 060-8638, Japan; 2 Department of Ophthalmology, Teine Keijinkai Hospital, Sapporo 006-8555, Japan

**Keywords:** deep learning-based pachychoroid index, pachychoroid neovasculopathy, neovascular age-related macular degeneration, artificial intelligence

## Abstract

**Background/Objectives**: To investigate the impact of pachychoroid on the clinical features of neovascular age-related macular degeneration (nAMD) in Japan using the deep learning-based Hokkaido University pachychoroid index (HUPI), which has a high discriminative ability for pachychoroid. **Methods**: This retrospective observational study examined 124 eyes of 111 treatment-naïve nAMD patients, including 44 eyes with type 1 macular neovascularization (MNV), 26 eyes with type 2 MNV, and 54 eyes with polypoidal choroidal vasculopathy (PCV). HUPI was calculated for each eye from EDI-OCT choroidal images using our modified LeNet that had learned the image patterns of pachychoroid. Differences in HUPI between nAMD types and inter-relationships between nAMD parameters, including HUPI, were evaluated. **Results**: The mean HUPI was 0.53 ± 0.30 for type 1 MNV, 0.33 ± 0.23 for type 2 MNV, and 0.61 ± 0.3 for PCV, with significant differences between any two of the three groups (*p* < 0.05, for each). Round-robin multiple regression analysis for nAMD parameters showed the close associations of the HUPI with choroidal vascular hyperpermeability (CVH) and subretinal fluid (SRF) (*p* = 0.017 and *p* < 0.001 for each) and the clear division of nAMD parameters into the following two groups: one including intraretinal fluid and type 1 and type 2 MNV and the other including SRF, CVH, polypoidal lesions, and HUPI. **Conclusions**: HUPI revealed that eyes with type 1 MNV and PCV had more pachychoroid-like features than eyes with type 2 MNV. HUPI was tightly associated with CVH and SRF but not MNV per se in nAMD parameters, reinforcing the pathoetiological concept of differentiating pachychoroid from typical nAMD.

## 1. Introduction

Pachychoroid is a common feature of diseases, including central serous chorioretinopathy (CSC), polypoidal choroidal vasculopathy (PCV), pachychoroid pigment epitheliopathy (PPE), and pachychoroid neovasculopathy (PNV), which are referred to as pachychoroid spectrum disease (PSD) [1,2,3,4]. CSC, a representative disease of PSD, and PPE [2], further complicated by retinal pigment epithelium (RPE) abnormalities, are known to induce macular neovascularization (MNV), which is clinically termed PNV [1], and the PNV often progresses to PCV. The prevalence of neovascular age-related macular degeneration (nAMD) in individuals aged 40 years and over has been reported to be 0.36% [5]. It has been reported that nearly half of Japanese patients previously diagnosed as nAMD conformed to the criteria of PNV [6,7]. The continuity between pachychoroid and MNV, as well as the high proportion of PNV in nAMD, make the concept of pachychoroid increasingly important as the pathogenetic background of nAMD, in addition to the conventional drusen and RPE abnormalities. However, the impact of pachychoroid on the clinical findings and pathogenesis of nAMD is unknown.

Since PNV was proposed as a new disease entity [1,2], there has been an increasing trend to classify nAMD into pachychoroid-driven or drusen-driven subtypes, considering these pathological conditions, while they are clinically classified into type 1–3 MNVs, and PCV as subtypes of type 1 MNV [8]. While drusen-driven nAMD can develop into any type 1–3 MNV, pachychoroid-driven nAMD, i.e., PNV, predominantly manifests type 1 MNV [9]. The lack of a broad consensus on the key concept of pachychoroid may complicate classifying nAMD and could potentially hinder clinical research. In particular, choroidal thickening per se has diminished its importance as the fundamental feature of pachychoroid compared to other choroidal vascular morphologies, such as pachyvessel and choroidal vascular hyperpermeability (CVH) [9]. Moreover, it is important to note that these choroidal vascular morphologies, such as pachyvessel and CVH, are qualitative features and difficult to define as numerical parameters.

Analyses using deep learning have become an indispensable part of various fields of ophthalmology [6,10,11,12,13,14,15]. We have previously developed the artificial intelligence (AI)-based “Hokkaido University pachychoroid index (HUPI)” learning from enhanced-depth imaging optical coherence tomography (EDI-OCT) choroidal images of CSC eyes. HUPI can quantify the pachychoroid-like features with a probability ranging from 0 to 1 (1: the most pachychoroid-like feature), and it has demonstrated excellent discriminative ability in differentiating choroidal images between acute-phase CSC and normal eyes (sensitivity 0.84, specificity 0.77, area under the receiver operating characteristic curve 0.86) [16].

In this study, using our novel AI-based pachychoroid index, HUPI, we investigated the impact of pachychoroid on clinical features of nAMD and reveal the cause–effect inter-relationships between them.

## 2. Materials and Methods

### 2.1. Study Subjects

This retrospective observational study followed the principles of the Declaration of Helsinki. The institutional review board of Teine Keijinkai Hospital (IRB number: 2-024079-00) approved this study on an opt-out basis, in which patients were given the opportunity to refuse to participate in the study via the website, since this was a non-invasive retrospective observational study. EDI-OCT images of 124 eyes of 111 Japanese patients diagnosed with treatment-naïve nAMD at Teine Keijinkai Hospital between August 2014 and May 2018 were enrolled in this study to investigate correlations between our deep learning-based pachychoroid index and the clinical features of nAMD.

### 2.2. Ophthalmic Examinations

All participants underwent a medical history inquiry, comprehensive clinical examinations, and ophthalmic examinations, including best-corrected visual acuity (BCVA), intraocular pressure, slit-lamp microscopy, fundus examination, and ocular imaging. The horizontal scan images of EDI-OCT (ZEISS CIRRUS HD-OCT 5000 with AngioPlex, Carl Zeiss Meditec, Inc., Dublin, OH, USA) at the initial visit without any treatment for nAMD were used. Decimal BCVA data were collected from the medical records on the same day as the OCT images were taken and were converted to logMAR BCVA. nAMD eyes were classified into type 1 MNV, type 2 MNV, and PCV by two experienced ophthalmologists based on funduscopic findings and OCT images obtained on the same day.

The exclusion criteria were as follows: (1) patients with a history of previous topical drug treatment for nAMD including intravitreal injections of anti-vascular endothelial growth factor (VEGF) agents, and (2) patients with intraretinal fluid (IRF) or subretinal fluid (SRF) due to other retinal diseases, including diabetic macular edema, retinal vein occlusion, or idiopathic macular telangiectasia.

### 2.3. The Neural Network for Our Deep Learning-Based Pachychoroid Index

A modified LeNet neural network using Python version 3.7.0 and NNabla version 1.33.1 (Sony Corporation, Tokyo, Japan) was trained and validated using 111 and 120 choroidal images (128 × 128 pixels), which were extracted from the EDI-OCT images of 37 representative CSC and 40 normal eyes, respectively, and named “Hokkaido University pachychoroid index” (HUPI), according to a previous report [16]. The HUPI quantifies the pachychoroid-like features of a choroidal image pattern on a probability ranging from 0 to 1 (1: the most pachychoroid-like feature). The cut-off value of HUPI to discriminate pachychoroid or not was calculated to be 0.66, with a sensitivity of 0.84 and a specificity of 0.77 [16].

### 2.4. Endpoints and Statistical Analyses

The primary endpoint to investigate HUPI in the characteristics of nAMD was the correlations between HUPI and 3 types of eyes with nAMD: type 1 MNV, type 2 MNV, and PCV. Type 2 MNV eyes were diagnosed even with type 1 MNV as well (i.e., mixed type 1 and type 2 MNV); PCV eyes were diagnosed as having polypoidal lesions associated with type 1 MNV.

In addition, the correlations between HUPI and clinical nAMD parameters, including age, sex, logMAR BCVA at the initial visit, presence or absence of SRF and IRF on EDI-OCT, CVH on indocyanine green angiography (ICGA), and structural MNV findings (type 1 MNV, type 2 MNV, and polypoidal lesions on EDI-OCT and ICGA) were examined. SRF, IRF, CVH, and MNV findings were used on a basis of ‘parameters’ but not ‘eyes,’ because these anatomical parameters frequently coexisted in a single eye. CVH findings were assessed for 100 of the 124 eyes in which ICGA was taken.

Statistical tests were performed using the R statistical package (version 3.6.1, R Foundation for Statistical Computing, Vienna, Austria). The Kruskal–Wallis test and Mann–Whitney U test corrected by Holm’s method as a post hoc were used to analyze differences in HUPI between the 3 types of nAMD. Mann–Whitney U test was used to analyze the differences in HUPI between the absence or presence of CVH and SRF. Simple linear regression analysis and multiple stepwise linear regression analysis determined the explanatory variables affecting HUPI and the explanatory variables affecting clinical nAMD parameters, including HUPI, respectively. *p* values less than 0.05 were considered statistically significant in all analyses.

## 3. Results

### 3.1. Clinical Background

**Figure 1:**
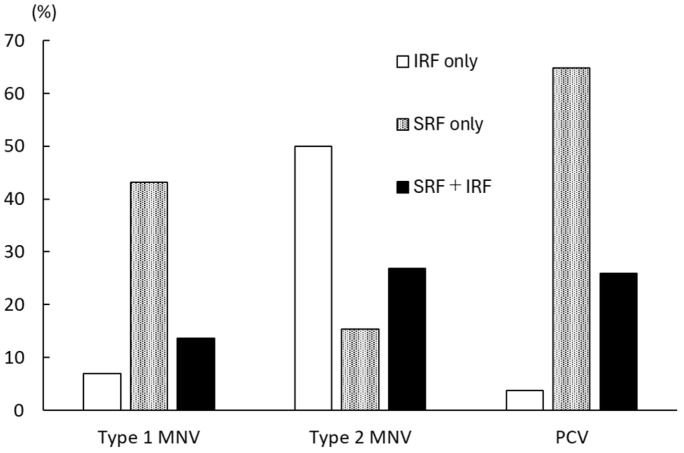
Incidence of retinal fluid subtypes among the types of neovascular age-related macular degeneration. Macular neovascularization, MNV; polypoidal choroidal vasculopathy, PCV; intraretinal fluid, IRF; subretinal fluid, SRF.

Table 1 shows the clinical background of 111 treatment-naïve Japanese nAMD patients (75.9 ± 8.6 years, 71 males and 40 females) with 124 eyes enrolled in the study. In total, 44, 26, and 54 eyes were classified into type 1 MNV, type 2 MNV, and PCV groups, respectively. As shown in Figure 1, the incidence of eyes with IRF only was 6.9% for type 1 MNV, 50.0% for type 2 MNV, and 3.7% for PCV. The incidence of eyes with SRF only was 43.2% for type 1 MNV, 15.4% for type 2 MNV, and 64.8% for PCV. The incidence of eyes with both IRF and SRF was 13.6% for type 1 MNV, 26.9% for type 2 MNV, and 25.9% for PCV.

### 3.2. Comparison of HUPI Between Types of nAMD

**Figure 2:**
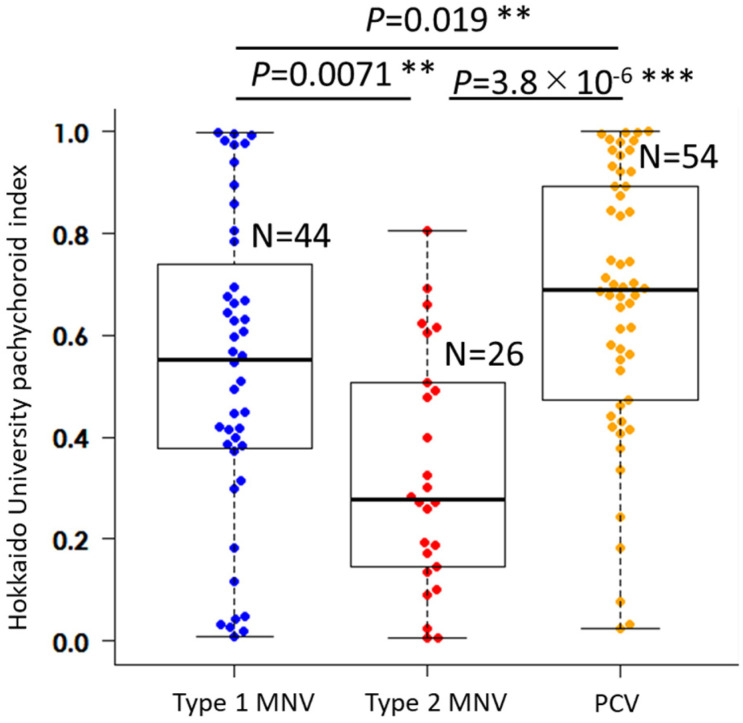
Comparison of “Hokkaido University pachychoroid index” (HUPI) among the types of neovascular age-related macular degeneration. Macular neovascularization, MNV; polypoidal choroidal vasculopathy, PCV; ** *p* < 0.01, *** *p* < 0.001, Mann–Whitney U test corrected by Holm’s method.

As shown in Figure 2, the mean HUPI was 0.53 ± 0.30, 0.33 ± 0.23, and 0.61 ± 0.30 for type 1 MNV, type 2 MNV, and PCV groups, respectively. There were significant differences among the three groups (*p* < 0.0001), i.e., type 1 MNV vs. type 2 MNV (*p* = 0.0071), type 1 MNV vs. PCV (*p* = 0.019), and type 2 MNV vs. PCV (*p* < 0.0001). The percentage of eyes with HUPI above the cut-off value of 0.66 was 27.3% for type 1 MNV, 7.7% for type 2 MNV, and 46.3% for PCV.

### 3.3. Analysis of Factors Contributing to HUPI

**Figure 3:**
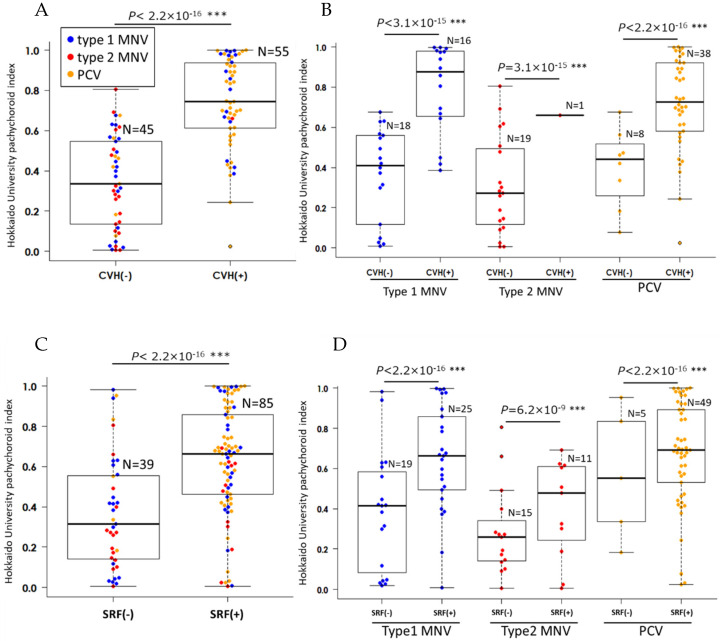
The difference in “Hokkaido University pachychoroid index” (HUPI) between neovascular age-related macular degeneration (nAMD) parameters associated with HUPI in multiple regression analysis. The box plot shows the HUPI of all types of nAMD eyes with and without choroidal vascular hyperpermeability (CVH) (**A**), and the HUPI of each type of nAMD with and without CVH (**B**). The box plot shows the HUPI of all types of nAMD eyes with and without subretinal fluid (SRF) (**C**), and the HUPI of each type of nAMD with and without SRF (**D**). Macular neovascularization, MNV; polypoidal choroidal vasculopathy, PCV; choroidal vascular hyperpermeability, CVH; subretinal fluid, SRF; blue dots, type 1 MNV; red dots, type 2 MNV; yellow dots, PCV. *** *p* < 0.001, Mann–Whitney U test.

As shown in Table 2, HUPI negatively correlated with age but positively with SRF, CVH, and polypoidal lesions (all: *p* < 0.001). Stepwise methods excluded age, sex, logMAR BCVA at the initial visit, IRF, polypoidal lesions, and type 1 and 2 MNVs from the explanatory variables. Multiple stepwise linear regression analysis revealed that CVH (*p* < 0.0001) and SRF (*p* = 0.017) significantly correlated with HUPI. Figure 3 shows the differences in HUPI between eyes with or without CVH and SRF, both of which were found to be associated with HUPI in multiple regression analysis. HUPI was significantly higher in eyes with CVH (Figure 3A,B) and SRF (Figure 3C,D), regardless of the types of nAMD (all: *p* < 0.001).

### 3.4. The Interactions Between Clinical nAMD Parameters

**Figure 4:**
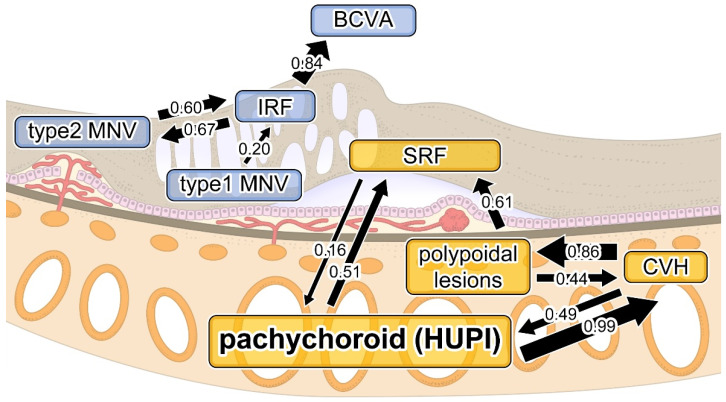
Cause–effect relationships between parameters by multiple regression analysis. The thickness of arrows reflects standardized regression coefficients (β values) for each pair of variables is related to the clinical nAMD parameters, which are divided into MNV (light blue) and pachychoroid (orange) clusters. “Hokkaido University pachychoroid index”, HUPI; macular neovascularization, MNV; choroidal vascular hyperpermeability, CVH; intraretinal fluid, IRF; subretinal fluid, SRF; best-corrected visual acuity, BCVA.

The inter-relationships between all nAMD parameters, including HUPI, were examined by multiple stepwise regression analyses of each item as an objective variable to identify explanatory variables affecting that particular objective variables. As shown in Table 3, logMAR BCVA positively correlated with IRF; SRF correlated with HUPI and polypoidal lesions; IRF correlated with type 1 and type 2 MNVs; CVH correlated with polypoidal lesions and HUPI; polypoidal lesions correlated with CVH; type 1 MNV did not correlate with any of the items; and type 2 MNV correlated with IRF. Furthermore, we illustrated in detail the inter-relationships among the characteristic findings of nAMD and HUPI. The results of the round-robin multiple regression analysis are shown in Figure 4. The thickness of the arrows indicates the values of standardized regression coefficients (β values) for each pair of explanatory and objective variables, between which the direction of arrows represents cause–effect relationships. The findings related to nAMD were divided into two major clusters, one including HUPI and CVH (Figure 4, orange background) and the other including MNV (Figure 4, light blue background).

## 4. Discussion

In this study, for the first time to our knowledge, we found several important findings regarding the impact of pachychoroid on the clinical features of nAMD as follows: (1) the mean HUPI was higher in the order of PCV, type 1 MNV, and type 2 MNV and was significantly different among the types of nAMD, and (2) the findings related to nAMD were divided into two major clusters, one including HUPI, CVH, and SRF and the other including MNV and IRF, with a significant association of HUPI with CVH and SRF, regardless of the types of nAMD.

Since the concept of pachychoroid was proposed [8], many researchers have investigated its impact on nAMD [17,18,19,20,21,22,23]. Pachychoroid is not a clearly delineated pathophysiology; rather, it transitions seamlessly between being drusen-driven and pachychoroid-driven in nAMD. Therefore, a numerical index representing the pachychoroid characteristics is needed to study its influence on nAMD. Conventional indices for quantifying pachychoroid-like features, such as central choroidal thickness (CCT) [17,18,19,20] and the luminal to total choroidal area ratio [21,22,23], are parameters designed based on the subjective human assessment of pachychoroid-like features, such as choroidal thickening and luminal enlargement. These indices do not utilize the full information available from image patterns within choroidal images. Thus, the insufficient discriminative power has limited the comprehensive analysis of the influence of pachychoroid on nAMD. The novel aspect of this study was that it utilized all the information in the OCT choroidal images by analyzing the pattern, which includes morphology, brightness, gradation, and other features using deep learning-based HUPI.

We elucidated that pachychoroid-like features were notably higher in the order of PCV, type 1 MNV, and type 2 MNV, showing significant differences among the types of nAMD. The percentage of eyes with HUPI greater than 0.66, the cut-off value to discriminate between CSC and normal eyes, was 27.3% for type 1 MNV, 7.7% for type 2 MNV, and 46.3% for PCV. In a previous report based on CCT in Japanese nAMD patients, pachychoroid was found in 58.3% of type 1 MNV, 14.0% of type 2 MNV, and 75.2% of PCV [7]. The nAMD phenotype is known to differ between Caucasians and Asians, and pachychoroid is more prevalent in Asians [24]. Hosoda et al. reported that 46.2% of Japanese nAMD patients exhibited pachychoroid-related features using deep phenotype unsupervised machine learning [6]. These results were consistent with the trend observed in the present study, which showed that a considerable proportion of nAMD had HUPI exceeding the cut-off value of pachychoroid, and the proportion was particularly high in type 1 MNV and PCV.

Furthermore, we illustrated in detail the inter-relationships among the characteristic findings of nAMD and HUPI. The findings related to nAMD were divided into two major clusters, one including HUPI and CVH (Figure 4, orange background) and the other including MNV (Figure 4, light blue background). HUPI was not associated with IRF, type 1 MNV, or type 2 MNV but was significantly associated with CVH and SRF, and the mean HUPI was significantly higher in cases with CVH and SRF, regardless of the types of nAMD (Figure 3). The tightest association of HUPI with CVH suggests that the calculation process of AI may reflect not only the dilation of the choroidal lumen and choroidal thickening in choroidal image patterns but also the leakage or accumulation of serous fluid into the choroidal stroma, which is the most essential pathogenesis of pachychoroid. The pathomechanism of pachychoroid directly causing SRF with little or no involvement of VEGF or MNV [9] has been widely discussed on the basis of low VEGF levels in PNV [25] and the association of complement factor H gene polymorphism susceptible to pachychoroid but not MNV or choroidal thinning [26,27]. Consistent with the previous studies, the broadly divided two clusters of nAMD features in our study suggested the weak association between pachychoroid and MNV, successfully distinguishing the pathomechanism of SRF, i.e., pachychoroid-driven, from the pathomechanism of MNV, i.e., drusen-driven. Recent research has demonstrated that the pathophysiology of pachychoroid is attributed to abnormal choroidal blood flow, with hyperperfusion resulting from increased sympathetic nerve activity [28,29,30,31] and congestion due to scleral thickening [32,33]. This differs fundamentally from the pathomechanism of MNV.

This study has some limitations. First, although we collected a relatively large number of nAMD participants, this was a retrospective observational study. A longitudinal cohort study is warranted to further validate our results showing the time course of nAMD. Second, the study included a limited number of patients with both eyes, which raises concerns about patient bias. Third, the correction of image magnification due to axial length and image tilt may further improve accuracy. However, this information is potentially contained in choroidal images. Fourth, a relatively simple neural network was used to maintain the robustness of the AI due to the small sample size, but a sufficient number of cases would allow for improved accuracy with a large neural network.

In conclusion, our deep learning-based pachychoroid index, HUPI revealed that eyes with type 1 MNV and PCV had more pachychoroid-like features than eyes with type 2 MNV. HUPI was tightly associated with CVH and SRF but not MNV per se in nAMD parameters, reinforcing the pathoetiological concept of differentiating pachychoroid from typical nAMD. As a clinically significant platform on the basis of pachychoroid pathophysiology, HUPI is likely to be useful for determining the timing and modalities therapeutic interventions against PSD and nAMD in future studies.

## Figures and Tables

**Table 1:** Demographic background and nAMD characteristics.

*Characteristics*	
**Age** (years)	75.9 ± 8.6
**Sex **(male: female)	71:40
**Type of MNV**	*n* (%)
Type 1 MNV	44 (35.5)
Type 2 MNV	26 (21.0)
PCV	54 (43.5)
**Subtype of retinal fluid**	*n* (%)
IRF only	58 (46.8)
SRF only	24 (19.4)
IRF + SRF	18 (14.5)

MNV, macular neovascularization; PCV, polypoidal choroidal vasculopathy; SRF, subretinal fluid; IRF, intraretinal fluid.

**Table 2:** Explanatory variables affecting “Hokkaido University pachychoroid index”.

	HUPI			
	Simple Linear Regression Analysis		Multiple Linear Regression Analysis	
	r	*p* Value	β	*p* Value
Age (years)	−0.31	**3.5 × 10^−4^**	-	-
Sex (male/female: 1/0)	0.045	0.62	-	-
LogMAR BCVA	−0.06	0.51	-	-
SRF	0.42	**1.1 × 10^−6^**	0.16	**0.017**
IRF	−0.11	0.21	-	-
CVH	0.64	**4.5 × 10^−13^**	0.49	**2.6 × 10^−6^**
Polypoidal lesions	0.34	**1.0 × 10^−4^**	-	-
Type 1 MNV	0.12	0.19	-	-
Type 2 MNV	−0.11	0.23	-	-

BCVA, best-corrected visual acuity; HUPI, Hokkaido University pachychoroid index; SRF, subretinal fluid; IRF, intraretinal fluid; CVH, choroidal vascular hyperpermeability; MNV, macular neovascularization; r, correlation coefficient; β, standardized regression coefficients. *p* < 0.05, statistically significant values are highlighted as bold.

**Table 3:** The interactions among clinical nAMD parameters.

Objective Variables	r	*p* Value	β	*p* Value
Explanatory Variables				
**LogMAR BCVA**				
IRF	0.46	**4.6 × 10^−8^**	0.84	**1.5 × 10^−5^**
**SRF**				
HUPI	0.42	**1.1 × 10^−6^**	0.51	**0.031**
Polypoidal lesions	0.41	**1.1 × 10^−6^**	0.61	**0.028**
**IRF**				
Type 1 MNV	0.11	0.22	0.2	**0.047**
Type 2 MNV	0.69	**2.2 × 10^−16^**	0.6	**0.0015**
**CVH**				
Polypoidal lesions	0.52	**1.9 × 10^−8^**	0.44	**0.0021**
HUPI	0.64	**4.5 × 10^−13^**	0.99	**4.6 × 10^−7^**
**Polypoidal lesions**				
CVH	0.52	**1.9 × 10^−8^**	0.86	0.06
**Type 1 MNV**				
Not detected	-	**-**	-	**-**
**Type 2 MNV**				
IRF	0.69	**2.2 × 10^−6^**	0.67	**3.5 × 10^−6^**

nAMD, neovascular age-related macular degeneration; BCVA, best-corrected visual acuity; HUPI, Hokkaido University pachychoroid index; SRF, subretinal fluid; IRF, intraretinal fluid; CVH, choroidal vascular hyperpermeability; MNV, macular neovascularization; r, correlation coefficient; β, standardized regression coefficients. *p* < 0.05, statistically significant values are highlighted as bold.

## Data Availability

The data that support the findings of this study are available on request from the corresponding author, M.S. The data are not publicly available due to their containing information that could compromise the privacy of the research participants.
